# Calcium Homeostasis and Bone Metabolism in Goats Fed a Low Protein Diet

**DOI:** 10.3389/fvets.2021.829872

**Published:** 2022-02-03

**Authors:** Hui Mi, Haobang Li, Weimin Jiang, Wu Song, Qiongxian Yan, Zhixiong He, Zhiliang Tan

**Affiliations:** ^1^CAS Key Laboratory for Agro-Ecological Processes in Subtropical Region, National Engineering Laboratory for Pollution Control and Waste Utilization in Livestock and Poultry Production, Hunan Provincial Key Laboratory of Animal Nutritional Physiology and Metabolic Process, Institute of Subtropical Agriculture, The Chinese Academy of Sciences, Changsha, China; ^2^University of Chinese Academy of Sciences, Beijing, China; ^3^Hunan Institute of Animal and Veterinary Science, Changsha, China; ^4^Hunan Co-innovation Center of Animal Production Safety (CICAPS), Changsha, China

**Keywords:** low-protein diets, bone metabolism, plasma Ca, metabolism biomarkers, goats

## Abstract

The objective of this study was to investigate the effects of low-protein diets on blood calcium (Ca) level, bone metabolism, and the correlation between bone metabolism and blood calcium in goats. Twenty-four female *Xiangdong* black goats with similar body weight (19.55 ± 3.55 kg) and age (8.0 ± 0.3 months) were selected and allocated into two groups: control group (CON, 10.77% protein content) and low-protein group (LP, 5.52% protein content). Blood samples were collected on days 1, 4, 7, 16 and 36 before morning feeding to determine the concentration of calcium (Ca), parathyroid hormone (PTH), bone gla protein (BGP), C-terminal telopeptide of type 1 collagen (CTX-1), bone alkaline phosphatase (BALP), and 1, 25-dihydroxyvitamin D3 [1,25(OH)_2_D3]. Liver samples were collected to determine the expression of bone metabolism-related genes. There was no difference observed between LP and CON in concentration of plasma Ca or any of bone metabolism markers (*P* > 0.05). In the liver, the mRNA expression of bone gamma carboxyglutamate protein (BGLAP), alkaline phosphatase (ALPL), and mothers against decapentaplegic homolog-1 (SMAD1) were increased (*P* < 0.05) in LP as compared with CON. The correlation analysis of Ca and bone metabolism markers showed no significant correlation between Ca and bone metabolism. These results suggest that the blood Ca concentration in mature goats may keep at a stable level through nitrogen cycling when the providing protein is not enough.

## Introduction

Calcium (Ca) is an essential mineral in the physical and is closely related to various physiological and pathological processes (1). It has been reported that bone metabolism is closely linked to Ca homeostasis, including bone resorption and bone formation, of which bone resorption is necessary to maintain an appropriate level of blood Ca (2). Bone is the calcium repository, containing about 98% of the total body calcium (3). Bone is a highly dynamic organ that is constantly metabolized and remodeled throughout life because bone plays a crucial role in providing support, maintaining mineral balance, and protecting soft tissue for the body (4, 5). The metabolic balance of bones depends on bone formation by osteoblasts and bone resorption by osteoclasts (6). Generally, biochemical markers of bone turnover have proved to be a valuable and simple tool for studying bone metabolism status (7), such as bone alkaline phosphatase (BALP), bone gamma-carboxyglutamate protein (BGLAP), and cross-linked C-terminal telopeptides of type I collagen (CTX-1). Moreover, bone development is profoundly influenced by signaling pathways, such as the Wingless tail (Wnt) signaling pathway (8), the OPG / RANKL / RANK signaling pathway (9), and the Smads signaling pathway (10). In addition, bone resorption dissolves the mineralized matrix of bone under the control of osteoclasts and hormones such as parathyroid hormone (PTH) and 1, 25-dihydroxyvitamin D3 (1,25(OH)_2_ D3), releasing ionized Ca into the blood and then altering the blood Ca concentration (11, 12). As most Ca is located in bone (13), and the plasma calcium concentration is tightly controlled in a narrow range to maintain numerous fundamental physiological functions of the body (14). In goats, it has been reported that the total plasma calcium ranges from 2.0 to 3.0 mmol/L (15), and Ca deficiency can lead to delayed growth and development and pathological risks such as osteoporosis, rickets, and osteochondrosis (16).

Dietary protein is essential for animal growth and health, and it is a vital source of synthetic osteoprotein amino acids (17, 18). However, resource shortage and ammonia emission are the main problems in protein feed application (19, 20). Thus, low-protein diets are commonly used to reduce feed costs and nitrogen emissions and maintain intestinal health (21, 22). Previous studies in young goats have demonstrated that severe mineral homeostasis and bone changes occurred in a reduced protein diet (23, 24). However, the effect of reduced dietary protein on Ca homeostasis and bone metabolism in mature goats is not yet fully understood. Therefore, we hypothesized that reducing dietary protein could alter the bone metabolic balance and regulate plasma Ca levels. Using mature goats as experimental animals, the objectives of this study were to determine the effects of a low-protein diet on bone markers (in the blood), bone metabolic gene expression (in the liver), plasma Ca concentration, and to evaluate the correlation between bone metabolism and blood Ca.

## Materials and Methods

The experiment was performed at the animal and crop laboratory building of the institute of subtropical agricultural ecology, Changsha, Hunan province. All experimental procedures were executed according to the Animal Care and the Use Guidelines of the Animal Care Committee, Institute of Subtropical Agriculture, Chinese Academy of Sciences, Changsha, China (ISA-2019-0115). All goats used in this study are purchased from Mulei Black Goats Breeding Farm in Liuyang, Changsha, China.

### Animal Management and Dietary Treatments

Twenty-four female *Xiongdong* black goats with a similar body weight of 19.55 ± 3.55 kg, about 8 ± 0.3 months old were used in this trial All goats were randomly assigned to either a control diet group (CON, 10.77% protein content, *n* = 12) or a low-protein diet group (LP, 5.52% protein content, *n* = 12). The protein maintenance requirements were the recommended values according to the Feeding Standard of Meat-producing Sheep and Goats of China (2004). The ingredients and nutrient composition of the experimental diets are shown in Table 1.

**Table 1:** Ingredients and compositions of the experimental diets.

**Item**	**CON^a^**	**LP^b^**
**Ingredients %**		
Rice straw	70.00	70.00
Soybean meal	15.00	0.00
Corn	8.20	23.00
Wheat bran	2.90	2.90
Calcium carbonate	0.10	0.10
Calcium biphosphate	0.30	0.50
Fat	1.00	1.00
sodium chloride	0.50	0.50
^c^Premix	2.00	2.00
**^d^Chemical compositions, %**		
Dry matter	96.17	95.86
Energy (MJ/kg)	16.76	16.84
Crude protein	10.77	5.52
Neutral detergent fiber	49.77	50.93
Acid detergent fiber	28.42	28.94

a *CON, control group;*

b *LP, low-protein diets group;*

c *Contained per kg of diet: 6.9 g FeSO_4_•7H_2_O; 4.6 g ZnSO_4_•H_2_O; 4.4 g CuSO_4_•5H_2_O; 11 g MnSO_4_•H_2_O; 104.2 g MgSO_4_•H_2_O; 0.3 g Na_2_SeO_3_; 11.2 g KI; 1.1 g CoCl2•6H_2_O; 15.4 g Vitamin.*

d *Chemical compositions were measured values*.

The total acclimatization period lasted for 14 days until all goats reached the stable dry matter intake. The experimental period lasted for 36 days. Each goat was housed in a single cage and fed twice daily (8:30 am and 5:30 pm) with the same amount of diet. All goats had free access to fresh water. A registered veterinarian slaughtered all goats with an intravenous injection of sodium pentobarbital (50 mg/kg BW) before feeding in the morning. All goats were weighed before being slaughtered at the end of the experimental period.

### Sample Collection and Analysis

Blood samples were collected from the jugular vein using vacuum blood collection vessels (containing anticoagulant) on days 1, 4, 7, 16, and 36 before morning feeding of the actual experimental period to investigate the periodic changes of bone metabolism markers. Plasma was obtained after centrifugation (2,500 × g, 10 min, 4°C) and then stored at −80°C until analyses. Plasma concentrations of 1,25(OH)_2_ D3, BGP, PTH, CTX-1, and BALP were measured using colorimetric methods with a spectro-photometer. The assay kits of PTH and BALP were purchased from MEIMIAN (Jiangsu, China). According to the manufacturer's specification, the assay kits of BGP and CTX-1 were purchased from MyBioSource Inc. (San Diego, SC, USA). The assay kits of 1,25(OH)_2_ D3 were purchased from Immundiagnostik AG (Bensheim, Germany). Plasma concentration of Ca was analyzed using a blood Ca concentration detection kit (Solarbio Life Sciences Co. Ltd., Beijing, China).

Liver samples were collected after slaughter to determine the gene expression that related to bone metabolism. Total RNA was extracted from liver tissue with the commercial kit (AG21017, Accurate Biology, Changsha, China), and then RNA was converted to cDNA using the RNA reverse transcription kit (AG11705, Accurate Biology, Changsha, China). The obtained cDNA template was stored at−80°C until real-time quantitative PCR analysis was performed.

### Real-Time Quantitative RT-PCR

Primers are shown in Table 2, designed with premier 5.0 software according to the relevant gene sequences of goat in Genebank. Real-time quantitative PCR was proceeded by ABI Prism 7900 HT Fast Real-Time PCR System (ABI, CA) with the SYBR® Premix Ex TaqTM II (Accurate Biology, China). The reaction system contained 5 μL SYBR® Premix Ex Taq TM (2x), 0.4 μL forward primer (10 μM), 0.4 μL reverse primer (10 μM), 0.2 μL ROX reference dye (50x), 1.0 μL cDNA, and 3 μL sterilized ddH_2_O under the amplification procedure (95°C for 10 min to active the DNA polymerase, then cycled at 95°C for 5 s, and 60°C for 30 s for 40 cycles). The specificity of products was determined according to dissolution curve under the dissolution procedure (95°C for 15 s, 60°C for 15 s and 95°C for 15 s).

**Table 2:** Primer sequences used for real-time quantitative PCR.

**Gene^a^**	**Primer sequences (5^′^-3^′^)^b^**	**Product** **size (Bp)**	**Gene bank**
ALPL	F: GAACCGATGTGGAGTATGAGC	110	XM_005677026.1
	R: GTGAGAGTGCTTGTGCTTCG		
BGLAP	F: GCAGCGAGGTGGTGAAGA	150	XM_013976665.1
	R: CTCCTGGAAGCCGATGTG		
VDR	F: CCACAAGACCTACGACGACA	130	XM_005680136.1
	R: GGAGGACGAGTTTCCAGAGA		
LRP5	F: ACGGCTCCGACGAACTCA	109	XM_013976027.1
	R: TGAAGAGGGACAAGATGATGC		
LRP6	F: AGGAGCGTCGTCAAGTAG	149	XM_005680825.2
	R: TGTAGGACCTGTGAGTGG		
β-catenin	F: TTACGGCAATCAAGAAAGCA	131	XM_005695574.1
	R: CAGACAGCACCTTCAGCACT		
MMP16	F: ACGGGCAGACCTTCGTATC	135	XM_005689258.1
	R: CATCACCCTGTGGTTTCTCA		
OPG	F: ATTTGGGCTCCTTCTAAC	103	XM_005689133.2
	R: CAGGGTCATGTCTATTCC		
RANKL	F: CTTTGCCCATCTCACGATTA	125	XM_005687420.1
	R: GTTTCCCATTGCTGAAGGTC		
BMP2	F: TAACTCTAAGATTCCCAAGGC	135	NM_001287564.1
	R: TAACGACACCCACAACCC		
SMAD1	F: ATCCCGAGTGGGTGTAGT	164	XM_013970504.1
	R: TCCTGGCGGTGGTATTCT		
GAPDH	F: TTCCACGGCACAGTCAAG	116	AJ431207.1
	R: TACTCAGCACCAGCATCACC		

a *ALPL, alkaline phosphatase, liver/bone/kidney; BGLAP, bone gamma carboxyglutamate protein; VDR, vitamin D (1, 25-dihydroxyvitamin D3) receptor; LRP5, low density lipoprotein receptor related protein 5; LRP6, low density lipoprotein receptor-related protein 6; β-catenin, beta-catenin; MMP16, matrix metalloproteinase 16; OPG, osteoprotegerin; RANKL, receptor activator for nuclear factor-κ B ligand; BMP2, bone morphogenetic protein 2; SMAD1, mothers against decapentaplegic homolog 1; GAPDH, glyceraldehyde-3-phosphate dehydrogenase.*

b *F, Forward primer; R, Reversed primer*.

Primers are shown in Table 2. The primer of GAPDH was designed online (https://www.sangon.com/newPrimerDesign), and others were referred to Li et al. (25). Relative quantification of the target gene expression was calculated from the melting curve and normalized against the abundance of the glyceraldehyde-3-phosphate dehydrogenase (GAPDH) gene, using the 2^−Δ*ΔCt*^ method (26), PCR and agarose gel electrophoresis verified all primers, and GAPDH has been tested across all tissue samples as a housekeeper gene.

### Statistical Analysis

All data were checked for normality and variance homogeneity before further statistical analysis. The data of blood Ca and bone markers required for ANOVA, the MIXED procedure of SAS 9.4 (SAS Inst. Inc., Cary, NC, USA) were used for analyzing, the model included the fixed effects of treatments, sampling time and their interaction, and the goat as a random effect. The bone, metabolism-related genes data was analyzed by an independent samples *t*-test using SAS 9.4 (SAS Inst. Inc., Cary, NC, USA). In addition, the Pearson correlation analyses were performed to calculate the correlation between blood Ca and bone metabolism markers. The significant difference was declared at *P* < 0.05, and the tendency was considered at 0.05 ≤ *P* < 0.10 for all statistical analyses.

## Results

### Ca and Bone Metabolism Markers in Plasma

**Figure 1:**
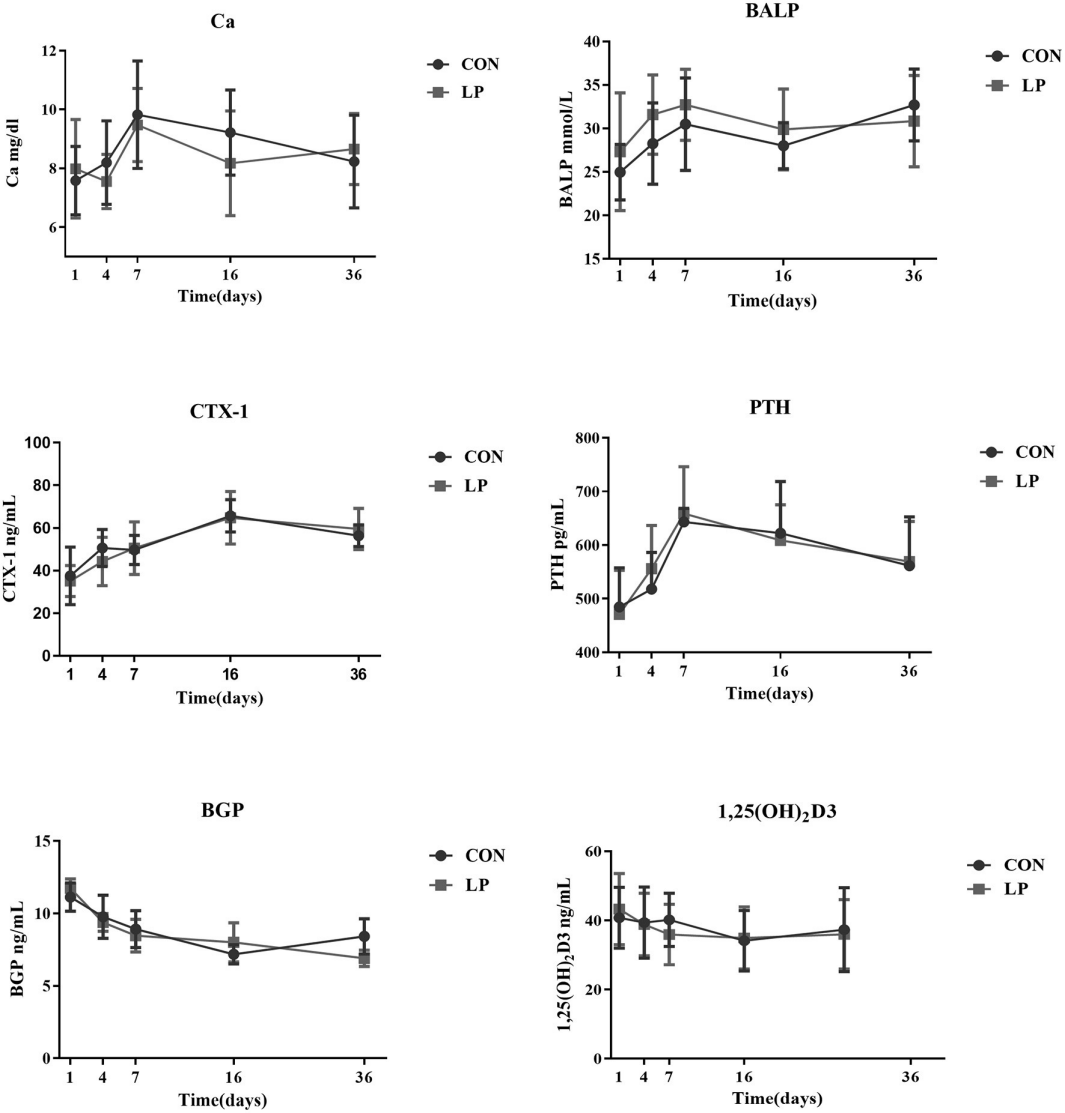
The effect of low protein diet on plasma calcium and bone metabolism markers of mature goats. Calcium, bone metabolism markers in Plasma. Ca, Plasma Calcium; CTX-1, bone resorption marker of C-terminal telopeptide of type 1 collagen; BALP, bone alkaline phosphatase; PTH, parathyroid hormone; BGP, bone gla protein; 1,25 (OH)_2_D3, 1, 25-dihydroxyvitamin D3. Each marker indicates the mean value, and the corresponding vertical bar indicates the standard deviation. N = 9 replicates in each group.

There was no difference in concentration of plasma Ca, bone markers, or any of hormones tested between LP and CON at any of the time sampled (*P* > 0.05, Figure 1).

### Expression of Bone Metabolism Related Genes in Liver

In the current study, the mRNA expression of BGLAP (*P* = 0.01), alkaline phosphatase, liver/bone/kidney (ALPL) (*P* = 0.037), and mothers against decapentaplegic homolog 1(SMAD1) (*P* < 0.01) in LP were higher than that from CON (Table 3). The lipoprotein receptor related protein 6 (LRP6) (*P* = 0.05), matrix metalloproteinase 16 (MMP16) (*P* = 0.07), and bone morphogenetic protein 2 (BMP2) (*P* = 0.06) mRNA expression in LP tended to be increased when compared with CON. However, the receptor activator for RANKL, low density lipoprotein receptor related protein 5 (LRP5), beta-catenin (β-Catenin), vitamin D (1, 25-dihydroxyvitamin D3) receptor (VDR), and OPG genes were not altered by the low-protein diet.

**Table 3:** The effect of low protein diet on gene expression in bone metabolism.

**Items^a^**	**Treat**		**SEM**	***P*-Value**
	**CON^b^**	**LP^c^**		
BGLAP	0.96	1.27	0.10	0.01
RANKL	1.75	2.04	0.33	0.11
MMP16	1.08	1.66	0.56	0.07
LRP6	1.14	1.87	0.45	0.05
ALPL	1.05	1.37	0.29	0.04
LRP5	1.05	1.26	0.33	0.18
β-Catenin	1.03	1.15	0.12	0.34
SMAD1	0.92	1.67	0.18	<0.01
BMP2	3.65	7.32	1.78	0.06
VDR	1.06	1.03	0.15	0.82
OPG	0.97	1.13	0.13	0.23

a *BGLAP, bone gamma carboxyglutamate protein; RANKL, receptor activator for nuclear factor-κ B ligand; MMP16, matrix metalloproteinase 16; LRP6, low density lipoprotein receptor-related protein 6; ALPL, alkaline phosphatase, liver/bone/kidney; LRP5, low density lipoprotein receptor related protein 5; β-catenin, beta-catenin; SMAD1, mothers against decapentaplegic homolog 1; OPG, osteoprotegerin; BMP2, bone morphogenetic protein 2; VDR, vitamin D (1, 25-dihydroxyvitamin D3) receptor.*

b *CON, control group, N = 11;*

c *LP, low-protein diet group, N = 9*.

### Correlation Analysis Between Ca and Bone Metabolism Markers

The correlation analysis showed that BGP was negatively correlated with CTX-1 (*r* = −0.55, *P* < 0.01) and BALP (*r* = −0.22, *P* = 0.05), while positively correlated with PTH (r = 0.43, *P* < 0.01). Meanwhile, CTX-1 was positively correlated with PTH (*r* = 0.31, *P* < 0.01), whereas no correlation (*P* > 0.05) was noted for the other variables (Table 4).

**Table 4:** Correlation analysis between calcium and bone metabolism markers^a^.

**Item^b^**	**CTX-1**	**BALP**	**PTH**	**BGP**	**1,25 (OH)_2_D3**
Ca	0.21	0.19	0.17	−0.02	0.10
	ns^c^	ns	ns	ns	ns
CTX-1		0.15	0.31	−0.55	−0.13
		ns	^**^	^**^	ns
BALP			0.13	−0.22	−0.03
			ns	^*^	ns
PTH				−0.43	−0.16
				^**^	ns
BGP					0.19
					ns

a *A relationship between plasma Ca and bone Metabolism markers.*

b *Ca, Plasma Calcium; CTX-1, bone resorption marker of C-terminal telopeptide of type 1 collagen; BALP, bone alkaline phosphatase; PTH, parathyroid hormone; BGP, bone gla protein; 1,25 (OH)_2_D3= 1, 25-dihydroxyvitamin D3.*

c *ns, non-significant;*

* *, P < 0.05 and*

** *, P < 0.01*.

## Discussion

This study investigated the changes of bone metabolism, and blood Ca concentrations in goats fed a low-protein diet. Numerous studies have supported the dietary protein involvement in regulating Ca metabolism by regulating kidney calcium emissions and intestinal calcium absorption, which ultimately manifests as changes in blood calcium concentration (27, 28). The current study showed no change in plasm Ca in goats fed a low protein diet. Similarly, it was reported that providing a low-protein diet did not affect Ca retention in healthy postmenopausal women (29). In a study using Sprague Dawley rats as the experimental animals, plasma concentrations of Ca were not altered when fed a low-protein diet (5% protein) as compared with the control group (20% protein) (30). Previous studies have shown that urine Ca production was positively related to Ca absorption, and Ca absorption and urine Ca excretion were in balance when dietary minerals met the demands (31). Therefore, the absolute amount of Ca retention was unaffected by reduced protein in our study, which resulted in a stable concentration in blood Ca. Moreover, it has been reported that blood Ca concentration in rats fed a low-protein diet was significantly decreased after 6 weeks but gradually returned to normal levels after 8 weeks (32), which was partly contradictory with our results. This difference would be ascribed to inconsistencies in the source of protein and experimental methodology (33). Meanwhile, different animal species may have various adaptations of the change of dietary protein levels. In goat studies, it has been reported that young male goats fed a reduced-protein for 6–8 weeks significantly reduced plasma Ca concentrations due to reduced intestinal Ca uptake and microbial metabolism (34–36), which was inconsistent with our results. The difference may be related with the different physiological state of the experimental goats gone through. In our study, we used the goats with an age of 8 months, which is considered an age of rumen-function well (37). In addition, we conducted the animal trial for 36 days, but no changes were observed between initial and final body weight in both CON and LP (final body weight: 19.97 vs. 20.23 kg when fed low-protein diet and control diet, respectively, unpublished data.), which may also indicate the goats we used are relatively mature. Because of the existing functional rumen, the nitrogen cycling mechanism of mature goats could provide N through microbial metabolism when the dietary protein is not enough. By possessing such efficient recycling mechanisms, mature goats could maintain rumen microbe's N supply and synthesize microbial protein as the source for host protein (34, 38, 39).

The current study showed that feeding a low protein diet had no change in bone metabolism markers in mature goats, including the markers reflecting the bone absorption and formation. These results were inconsistent with previous studies that a protein-restricted diet increased CTX-1 but decreased plasma 1,25(OH)_2_D3 concentrations in monogastric species and young small ruminants (23, 40). To better understand the bone metabolism mechanism of goats fed a low-protein diet, we measured the mRNA expression level of bone metabolism-related genes in liver, which is closely related to bone metabolism (25). It has been reported that BGLAP and ALPL, promote osteoblast activity and bone mineralization (41, 42). The increased gene expression of BGLAP and ALPL in LP indicated that the increased level of liver osteoblastic genes may compensate for the side effect from a low protein diet and keep the balance of bone turnover. Moreover, BMP2 and SMAD1 are the essential components of the BMP-Smad-Runx2 axis, which plays a vital role in bone development and formation (43). LRP6 plays a pivotal role in mediating bone cells and bone formation activity in the typical Wnt signaling pathway (44, 45). In our study, the low protein diet increased expression of BMP2, SMAD1 and LRP6. Therefore, it is possible that the goats with insufficient dietary protein may maintain the balance of bone metabolism through the BMP-Smad Runx2 axis and the Wnt signaling pathway.

The negative association between BGP and BALP was expected. In general, BALP is increased during osteoblast proliferation and extracellular matrix (ECM) maturation, and it is decreased during advanced mineralization (46, 47). In contrast, the BGP synthesis only occurs during mineralization (46, 47). The results showed that PTH was positively correlated with BGP and negatively correlated with CTX-1, which were expected. These results can be explained by the fact that PTH can stimulate bone resorption to regulate Ca homeostasis (11). Duque et al. (48) showed that the osteocalcin concentration was significantly increased in SAM-P/6 mice under the treatment of 1,25(OH)_2_ D3. This result was consistent with the positive correlation between BGP and 1,25(OH)_2_D3 observed in this experiment. There was no association between blood Ca and bone metabolism markers, which was inconsistent with the previous results obtained in monogastric species, such as using human, mice and chicken (49, 50). It has been shown that the blood Ca was regulated by exchange from bone, absorption in the intestine, and reabsorption in the kidney, which are all tightly correlated with PTH and 1,25(OH)_2_D3 (51). The lack of association between blood Ca and bone metabolism markers observed in our study may be due to the exist of rumen, which is engaged in calcium transport and absorptions. It has been reported that the Ca transport in the rumen are probably not regulated by 1,25(OH)_2_D3, nor mediated by the 1,25 (OH)_2_D3 related binding proteins and Ca channels (52). In terms of reabsorption in the kidney, ruminant renal seems insensitive to a dietary Ca restriction induced challenge of Ca homeostasis than monogastric animals (53, 54).

## Conclusion

In summary, no change was observed in concentrations of plasma Ca or bone metabolism markers in goats fed a low protein diet. Correlation analysis showed that no significant relationship between Ca and any bone metabolism markers. These results suggest that there may be compensatory homeostasis mechanisms in mature non-pregnant goats that maintain relatively stable bone metabolism markers and Ca concentration when feeding a low protein diet for a certain period of 36 days. Moreover, a low protein diet up-regulated the gene expression of BGLAP, ALPL and SMAD1, suggesting that the goats with insufficient dietary protein may maintain the balance of bone metabolism through the BMP-Smad Runx2 axis and the Wnt signaling pathway.

## Data Availability Statement

The raw data supporting the conclusions of this article will be made available by the authors, without undue reservation.

## Ethics Statement

The animal study was reviewed and approved by Animal Care Committee, Institute of Subtropical Agriculture, Chinese Academy of Sciences, Changsha, China.

## Author Contributions

QY: methodology. HM, HL, WJ, and WS: animal experiment. HM: analysis, data curation, and writing—original draft preparation. ZH and ZT: writing—review and editing. ZH: funding acquisition. All authors have read and agreed to the published version of the manuscript.

## Funding

This work was jointly supported by the Hunan Key Research and Development Program (2020NK2049) and Innovation Province Project (2019RS3021).

## Conflict of Interest

The authors declare that the research was conducted in the absence of any commercial or financial relationships that could be construed as a potential conflict of interest.

## Publisher's Note

All claims expressed in this article are solely those of the authors and do not necessarily represent those of their affiliated organizations, or those of the publisher, the editors and the reviewers. Any product that may be evaluated in this article, or claim that may be made by its manufacturer, is not guaranteed or endorsed by the publisher.
